# Advancements in Wearable EEG Technology for Improved Home-Based Sleep Monitoring and Assessment: A Review

**DOI:** 10.3390/bios13121019

**Published:** 2023-12-07

**Authors:** Manal Mohamed, Nourelhuda Mohamed, Jae Gwan Kim

**Affiliations:** Biomedical Science and Engineering Department, Gwangju Institute of Science and Technology, Gwangju 61005, Republic of Korea; manalalnosh@gm.gist.ac.kr (M.M.); nonoalhodaali@gm.gist.ac.kr (N.M.)

**Keywords:** EEG, wearable, sleep monitoring

## Abstract

Sleep is a fundamental aspect of daily life, profoundly impacting mental and emotional well-being. Optimal sleep quality is vital for overall health and quality of life, yet many individuals struggle with sleep-related difficulties. In the past, polysomnography (PSG) has served as the gold standard for assessing sleep, but its bulky nature, cost, and the need for expertise has made it cumbersome for widespread use. By recognizing the need for a more accessible and user-friendly approach, wearable home monitoring systems have emerged. EEG technology plays a pivotal role in sleep monitoring, as it captures crucial brain activity data during sleep and serves as a primary indicator of sleep stages and disorders. This review provides an overview of the most recent advancements in wearable sleep monitoring leveraging EEG technology. We summarize the latest EEG devices and systems available in the scientific literature, highlighting their design, form factors, materials, and methods of sleep assessment. By exploring these developments, we aim to offer insights into cutting-edge technologies, shedding light on wearable EEG sensors for advanced at-home sleep monitoring and assessment. This comprehensive review contributes to a broader perspective on enhancing sleep quality and overall health using wearable EEG sensors.

## 1. Introduction

Sleep plays a pivotal role in our daily lives, and its significance cannot be ignored. It is a fundamental part of our routine, impacting every organ and system within our body including the brain, heart, immune system, and cellular metabolism. Maintaining a consistent daily sleep schedule is essential for overall health and well-being.

Inadequate sleep quality has far-reaching consequences across various aspects of our lives. It adversely affects our physical, cognitive, emotional, hormonal, mental, and cardiac health as well as our daily physical activities and overall sense of well-being. Scientific evidence has established a clear link between insufficient sleep duration and a range of serious health conditions, including hypertension [1], cardiovascular diseases [2,3], diabetes [4], obesity [5], psychiatric illnesses [6], and neurodegenerative diseases such as multiple sclerosis [7] and Alzheimer’s disease [8].

Despite the critical role of sleep in our health, many individuals struggle to maintain high-quality sleep patterns [9]. Shockingly, 18% of the United States population sleeps less than 6 h per day. This deficiency in sleep quality leads to a multitude of health issues and a decline in productivity, resulting in a staggering economic loss of approximately USD 411 billion, with projections indicating an increase to USD 467 billion by 2030 [10]. To mitigate these challenges and safeguard our health, it is important to implement effective and continuous sleep-monitoring practices [11].

**Figure 1:**
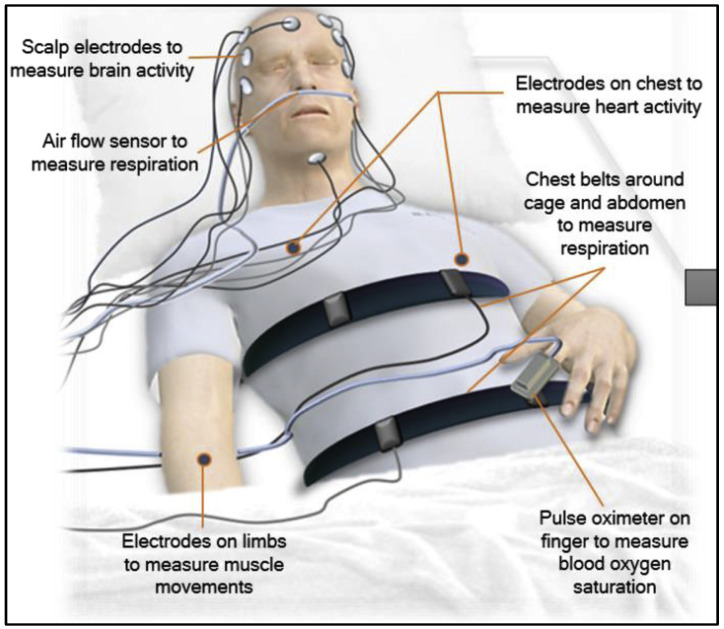
Set-up of the polysomnography (PSG) and the signals that could be recorded using it [17].

Polysomnography (PSG) is the most commonly used method for sleep monitoring in hospitals and medical centers, recording a variety of physiological signals related to sleep, such as electroencephalogram (EEG), electrooculogram (EOG), electrocardiogram (ECG), and electromyogram (EMG) [12,13,14,15], alongside numerous other signals, as illustrated in Figure 1. Generally, sleep experts are engaged in meticulous manual annotation and identification of specific signal patterns. This process entails segmenting the overall PSG recording into 30 s epochs with adherence to the guidelines established by the American Academy of Sleep Medicine (AASM) [16].

Despite the PSG device’s capability to simultaneously record a multitude of signals, it presents several notable drawbacks when it comes to long-term monitoring:

- Complex set-up: first and foremost, the installation process for PSG is highly intricate and time-consuming, often taking up to an hour for a skilled technician to complete.
- High cost: another significant drawback is its substantial cost [17,18,19], with expenses ranging from USD 1500 to USD 2000 per night in the United States.
- Inconvenient clinic visits: to undergo PSG monitoring, individuals are required to visit a sleep clinic and spend one or more nights there. This set-up often leads to an atypical night of sleep, which may not accurately represent their typical sleep patterns [7,19,20].
- Wired transducers and sensors: PSG necessitates the use of multiple wired transducers and sensors, resulting in a somewhat cumbersome and restrictive experience.
- Skilled technician requirement: the proper operation of PSG demands trained technicians who are proficient in its usage [17,21].
- Electrode fit assurance: lastly, there is a critical need to ensure that the electrodes are correctly and securely fitted [22], and this requires professional guidance.

To overcome the limitations associated with PSG, current research is increasingly emphasizing the use of wearable and portable devices for sleep monitoring, as evidenced by recent studies [23,24]. This shift is not only a response to PSG’s shortcomings, but also a reflection of the remarkable advancements in wearable technology. These technological advancements serve as a compelling catalyst, propelling scientists to delve deeper into this avenue.

Several review papers have been published, offering comprehensive insights into recently developed wearable sleep-monitoring systems, with a primary focus on their accuracy and effectiveness in sleep intervention. However, it is worth noting that most of these papers focus only on commercially available devices found on the market [13,25]. Also, there is a notable absence of review articles that specifically delve into the advancements in EEG technology for sleep monitoring [17,21,26]. For example, [26] describes the research progress of bioelectrical, biomechanical, and biochemical signals used for sleep monitoring without going into deep details about sleep-monitoring systems that acquire them. Also, it focuses on only a few EEG sleep-monitoring devices, providing limited insight into their design characteristics and properties. Furthermore, it does not conduct a thorough comparison with other wearable EEG sleep-monitoring systems. They mention those systems briefly in Section 3.1 of their paper.

Therefore, there should be review papers focusing on addressing wearable EEG technology for sleep monitoring, as this technology holds immense significance in the realm of sleep monitoring. EEG captures a wealth of information about brain activity during sleep, and it stands as the main indicator for determining sleep stages and identifying sleep disorders [16]. In contrast, systems based on other technologies, such as wristbands equipped with integrated photoplethysmography and motion sensors, are often lauded for their convenience. However, these systems fail to provide comprehensive coverage of the necessary physiological information essential for accurate and in-depth sleep analysis [27].

There is, however, a solitary review article that narrows its focus to headband-like wearable devices [28]; this particular article offers a thorough explanation and summary of both commercial and research-based headband sleep-monitoring systems. Nevertheless, it is important to note that their comparative analysis is somewhat limited, as it is primarily focusing on parameters gathered by these systems and their cost, while overlooking a more comprehensive comparison between these systems.

In this comprehensive review paper, our objective is to fill this gap by thoroughly examining the recent advancements in EEG wearable systems utilized for sleep monitoring. We will explore their capacity to provide convenient and precise sleep assessments, even within the comfort of one’s home. Furthermore, we will undertake an in-depth comparative analysis of these systems, shedding light on their respective strengths and weaknesses. Additionally, we will delve into the critical aspect of studies that assess and validate these systems, ensuring a well-rounded understanding of their performance and reliability.

## 2. Wearable EEG-Based Sleep-Monitoring Systems

Wearable EEG devices employed for sleep monitoring can be broadly categorized depending on their form factor into four main types: rigid headbands, flexible headbands, highly flexible EEG sleep-monitoring systems, and ear-EEG sleep-monitoring plugs and patches. Within each category, we will provide a detailed explanation of specific devices considering factors such as their structural design, materials used, and the methods employed for sleep assessment. This analysis is intended to offer valuable insights for designers, facilitating comparisons among these systems and aiding developers in their future endeavors.

Key aspects that will be scrutinized include EEG electrode positioning and quantity to ensure robust signal strength, EEG electrode type and material for optimal skin contact, EEG resolution and sampling rates, as well as the incorporation of EOG and chin EMG components to enhance overall accuracy. Table 1 provides a summary of recently developed wearable EEG systems used for sleep monitoring, all of which will be elaborated upon in this study.

### 2.1. Rigid Headbands

EEG-based sleep-monitoring systems often take the form of rigid headbands. These headbands exhibit varied shapes but share the common characteristic of being fixed, incorporating either wet or dry electrodes depending on their design. In this section, we will present three distinct rigid headbands, addressing the comparison between them in terms of design, advantages, and disadvantages.

#### 2.1.1. The Dreem Headband (DH)

The Dreem Headband (DH) is one of the commercial headbands used for sleep monitoring. Its widespread adoption in the scientific literature from 2019 up to now underscores its significance in sleep research. It represents a wireless, rigid headband designed to be worn during nocturnal hours, enabling the automatic real-time analysis of physiological sleep-related data. The DH is equipped to monitor five distinct categories of physiological activities, prominently brain activity. This activity is captured through five EEG dry electrodes strategically positioned across frontal (F7, F8, and Fpz) and occipital (O1 and O2) lobes, yielding seven derivations (Fpz–O1, Fpz–O2, Fpz–F7, F8–F7, F7–O1, F8–O2, and Fpz–F8). These electrodes operate at a sampling frequency of approximately 250 Hz, employing a bandpass filter spanning from 0.4 to 18 Hz.

**Figure 2:**
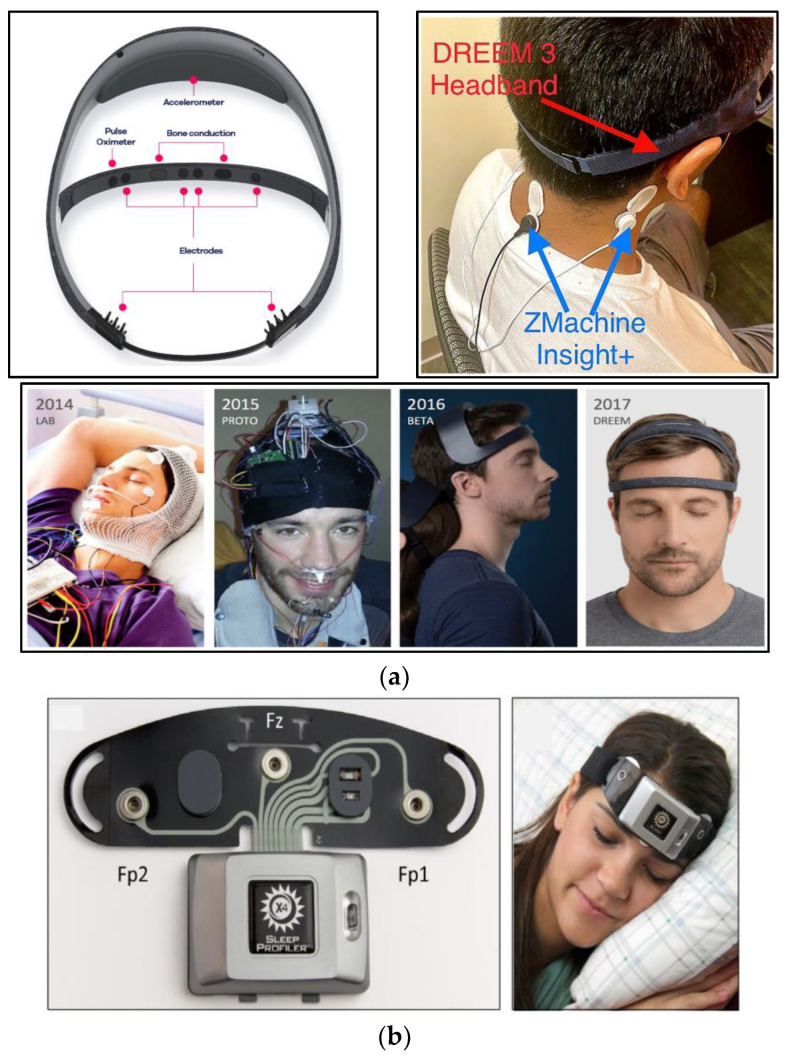

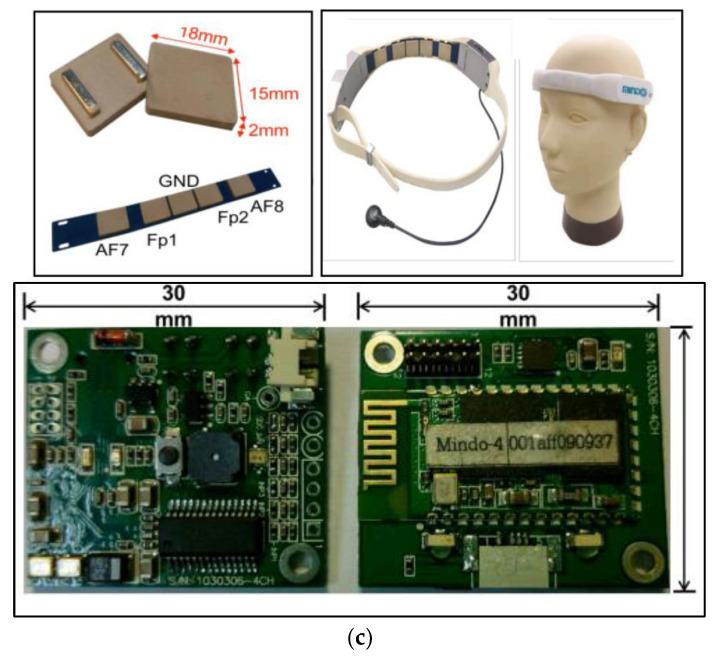
Rigid headbands. (**a**) The Dreem Headband (DH): (upper left) the configuration of sensor placements on the DH [29]; (lower) evolution of the DH [59]; and (upper right) simultaneous EEG signal recording with DREEM3 3 and Zmachine Insight+ [32]. (**b**) The Sleep Profiler (SP): (left) SP X4 with EEG electrode’s locations; and (right) SP X4 in use [35]. (**c**) Rigid headband with flexible EEG dry sensor: (upper left) silicon-based EEG sensor and placement of EEG channels; (lower) EEG acquisition circuit; and (upper right) wearable headband and forehead EEG sensors [38].

The DH has the ability to record a spectrum of physiological events, encompassing movement, position, breathing, and heart rate using a 3D accelerometer and a pulse oximeter [29]. The EEG electrodes situated at the DH’s frontal region are crafted from dry carbon-infused materials, characterized by high-consistency silicone composition. In contrast, the electrodes located at the rear portion possess an additional attribute distinguishing them from their frontal counterparts. This attribute involves soft and flexible protrusions, strategically designed to facilitate signal acquisition even through hair obstructions, as illustrated in Figure 2a (upper left).

As documented in the Dreem Whitepaper from 2019 [59], the evolution of the DH transpired across three distinct stages, culminating in the design depicted in Figure 2a (lower). Commencing in 2014, the endeavor started with the creation of an EEG headset intended to be used within laboratory environments. By 2015, the initial operational prototype had taken form. Progressing further, the beta iteration of the DH was realized in 2016. Finally, the pinnacle of this iterative process was achieved in 2017, marked by the attainment of the definitive DH design, beautifully showcased in Figure 2a (lower).

An automatic algorithm for sleep staging predictions is embedded in the DH. This algorithm operates through two crucial stages: the feature extraction stage and the classification stage. In the feature extraction stage, feature extraction takes place for each 30 s epoch, and many EEG features, including power frequency in the delta, alpha, theta, and beta bands, as well as the ratio of relative powers, are extracted and fed into the classification layer. Additionally, other features from the accelerometer and pulse oximeter data are also extracted to contribute to a total of 79 features for each raw DH record.

The classification stage includes two layers of long- and short-term memory and a softmax function, which are trained using backpropagation to predict the sleep stage to which the epoch belongs [29].

In 2019, an evaluation was undertaken to gauge the signal quality captured by the DH. To comprehensively assess its efficacy, a comparison was drawn against the conventional PSG, renowned as the benchmark for sleep monitoring [29]. The aim was to ascertain the DH’s overall performance. Notably, the results showcased a robust correlation between the EEG signals captured by the DH and those from PSG. This indicated the DH’s precision in accurately recording brain activity during sleep. Impressively, the DH’s automated sleep staging algorithms exhibited a level of accuracy comparable to that wielded by sleep experts. This compelling evidence suggests that the DH holds significant potential as a valuable tool for the execution of large-scale sleep studies.

Numerous studies have explored the viability and precision of the DH. In 2021, Kafashan and his colleagues conducted a study [30] aimed at assessing the DH’s capacity to capture perioperative sleep data in elderly cardiac surgical patients. The research determined that the DH was comfortably wearable during sleep for the patients. Among the 90 patients invited to partake in the study, an impressive 74 (or 82%) were able to successfully record at least one night of sleep data through the DH prior to their surgical procedures. This accomplishment becomes even more notable when considering the diverse geographical origins of the patients and the relatively brief time window before their surgeries. The quantity of sleep data collected was deemed substantial, given these circumstances. Typically, patients contributed data from one night, and it was observed that half of the participants recorded between 0 and 2 nights of sleep data.

Another investigation was conducted in 2022 by Zambelli and his team [31] to ascertain the feasibility and acceptability of the updated iteration of the original DH, known as DREEM 2, within the context of adults suffering from chronic pain. The findings of this study were enlightening, as 90% of the 21 participants were able to record at least two nights of sleep data using the headband. Remarkably, each participant contributed data from at least one night; 76% of the participants were satisfied with the study, and 86% were willing to wear the headband for longer than the minimum requirement of two nights. Additionally, 76% found the headband comfortable while awake, and 57% found it comfortable while sleeping. The study concluded that the DREEM 2 headband is a feasible and acceptable way to collect sleep data among individuals with chronic pain, even though they often experience sleep disturbances.

In December 2022, Wood and his colleagues spearheaded a study [32] aimed at assessing the capabilities of the DREEM 3, an advanced iteration of the original DH, in tandem with another widely employed sleep-monitoring tool named Zmachine Insight+. Like the DH, the Zmachine Insight+ utilizes EEG technology to monitor sleep patterns and is renowned for its user-friendly nature, necessitating minimal training. Within this investigation, sleep data from 25 subjects were simultaneously recorded using both devices and kept recording it in a sleep log, as shown in Figure 2a (upper right). They discovered that the Zmachine Insight+ tended to overestimate wakefulness durations, whereas the DREEM 3 exhibited a tendency to underestimate them. Notably, the agreement between these two devices displayed an enhancement from the initial night to the subsequent one. Armed with these findings, the study put forth recommendations to optimize the utilization of these devices. For the DREEM 3, it was suggested that users employ a sweatband over the device to prevent potential movement during sleep. Furthermore, in situations where both accuracy in sleep scoring and practical functionality are pivotal considerations, the study underscored the DREEM 3 as the favored option. This recommendation is particularly pronounced when sleep staging holds paramount importance for researchers, especially within populations of young, healthy individuals.

Nowadays, many studies use the DH as the primary reference for assessing sleep–wake patterns and sleep stages against various other commercially available devices. For instance, a study conducted by Chinoy and colleagues [60] evaluated the performance of four commercial wearable sleep-tracking devices (Fatigue Science Readiband, Fitbit Inspire HR, Oura ring, and Polar Vantage V Titan) in comparison to the DREEM 2 in a home setting.

#### 2.1.2. The Sleep Profiler (SP)

Another sleep-monitoring headband that has been widely used in the literature is the Sleep Profiler (SP), which is also a wireless rigid headband designed for sleep monitoring. Its enhanced version, the SP X4, has been employed in numerous research studies. This version is equipped with three frontopolar EEG electrodes (AF7, AF8, and Fpz) for EEG data acquisition [34,35], as depicted in Figure 2b. Additionally, it can capture various physiological activities beyond EEG, such as pulse rate and head movement and position using a 3D accelerometer. These physiological activities, including EEG, are transferred to an online software platform known as the SP Portal. Within this portal, each data entry undergoes assessment through an automated staging algorithm named SP Auto, which is designed under the general staging rules outlined by the AASM [34].

In 2017, Daniel and his colleagues conducted an assessment of the SP’s reliability in measuring sleep architecture and sleep continuity biomarkers [36]. Their study revealed a robust correlation between automated sleep staging and human-scored PSG. A single night of recording was sufficient to identify abnormal slow-wave sleep, sleep spindle activity, and heart rate variability among patients. However, a two-night recording yielded a more comprehensive evaluation of other sleep biomarkers.

Another study by Patrick and his associates [34] scrutinized the SP’s accuracy. The findings demonstrated that the SP is precise compared to the gold standard PSG system. Notably, the SP accurately discerned various sleep stages, excelling particularly in Stage N3 and REM sleep classification.

In the same year, Brendan and his colleagues explored the viability of SP’s sleep scoring using a single EEG channel in comparison with PSG [35]. The outcomes affirmed the SP’s accuracy and parity with PSG, albeit with some limitations. The study concluded that the single-channel EEG device could serve as a valuable research tool for evaluating REM sleep and several other parameters.

Furthermore, multiple studies have harnessed the SP in investigating sleep-related disorders. For instance, a study [37] evaluated the accuracy of a sleep staging system in patients with isolated REM sleep behavior disorder (iRBD). This study compared physicians’ diagnoses of iRBD based on REM sleep without atonia (RSWA) and non-REM hypertonia (NRH) using data collected from both SP and PSG recordings.

#### 2.1.3. Rigid Headband with Flexible EEG Dry Sensor

The DH and SP are commercially available rigid headbands that have garnered extensive usage in the scientific literature. By acknowledging them, we could have a solid comparative reference with other systems. Now, we will introduce a distinct category of headband—a noncommercial rigid headband. This specific headband, detailed in reference [38], was designed by Chin and his colleagues. They focused on creating a sleep-monitoring system using dry electrodes instead of wet ones aiming to overcome the limitations of wet electrode-based systems, which are uncomfortable and time-consuming. Also, the wet electrode’s gel can dry out resulting in reduced EEG signal quality and long-term monitoring reliability. Consequently, the adoption of dry electrodes could overcome these limitations, simplify EEG signal acquisition, and help bridge the gap between neuroscience research or clinical practice and real-life applications.

This group of researchers initiated their work by creating an innovative flexible dry electrode made from silicon [61]. The fabrication process involved using silicon, silver, AgSiO_2_, gel, and thick-film pastes. Through precise proportions of these components, they successfully developed an electrode with dual advantages: low impedance and outstanding flexibility. These electrodes are very light in weight and utilize limited materials to enhance sensitivity.

The designed electrodes are notably compact with a size of about 18 mm × 15 mm × 2 mm, and they find their placement at strategic positions: AF5, Fp1, Fp2, and AF8, all within the forehead band. This band accommodates two circuit boards that comprise the EEG acquisition circuit, and it is about 30 mm × 25 mm × 5 mm in size. The complete design of the system is visually depicted in Figure 2c.

The designed system can automatically classify sleep stages through three steps: preprocessing, feature extraction, and classification. During the preprocessing step, the acquired data undergo down-sampling, filtering, and short-time Fourier transformation to capture the spectral patterns in the EEG. In the feature extraction step, EEG features are extracted from each of the following bands: low delta, delta, theta, sigma, beta, and gamma. This results in 13 features fed into the third and final step—the classification step, which employs a relevance vector machine (RVM) that effectively categorizes the five sleep stages [38].

Table 2 provides a comparison of the three aforementioned rigid headbands. The DH has the highest number of channels compared to both X4 SP and the forehead headband mentioned in [38], while SP offers extended recording time, but their usage of wet electrodes can lead to dehydration, thereby adversely affecting the quality of the EEG signals. On the other hand, the forehead headband mentioned in [38] emerges as a superior option, as it employs dry electrodes instead of wet ones.

As stated in Table 1, both DH and SP capture not only EEG but also other physiological parameters, allowing for a more comprehensive understanding of the subject’s sleep and rendering both DH and SP more advantageous compared to the forehead headband mentioned in [38].

It is worth noting that all three systems are rigid headbands that have several disadvantages, such as causing discomfort when worn and potentially disrupting sleep, resulting in data recorded in an unusual sleep environment. Moreover, the bulky and inflexible design of these headbands discourages users from wearing them during sleep. Given these limitations, recent research has been exploring alternative form factors for sleep EEG monitoring systems, which will be discussed in the following sections.

### 2.2. Flexible Headbands

This category of headbands possesses the characteristic of being more flexible than rigid headbands, leading to optimal attachment, acquisition of high-quality EEG signals, and user comfort.

#### 2.2.1. Flexible Headband with Silver-Coated Fabric Dry Sensor

In 2012, prior to the development of DH, SP, and the forehead headband mentioned in [38], Shambroom and his research team pioneered a system [39] that exhibited a certain degree of flexibility compared to rigid headbands. Their aim was to evaluate a new wireless sleep-monitoring system that could automatically record and score human sleep without the need for skilled personnel.

The wireless system they conceptualized is a headband with dry silver-coated fabric sensors strategically positioned to capture EEG signals from a single bipolar channel located at the forehead region (Fp1–Fp2). Inside the headband, there is an analog-to-digital converter and a processing unit to amplify and filter the acquired signals. An ultra-low-power priority protocol operating at 2.4 GHz is employed to transmit the processed signal to a designated base station equipped with a microprocessor that utilizes an artificial neural network for calculating sleep stages.

**Figure 3:**
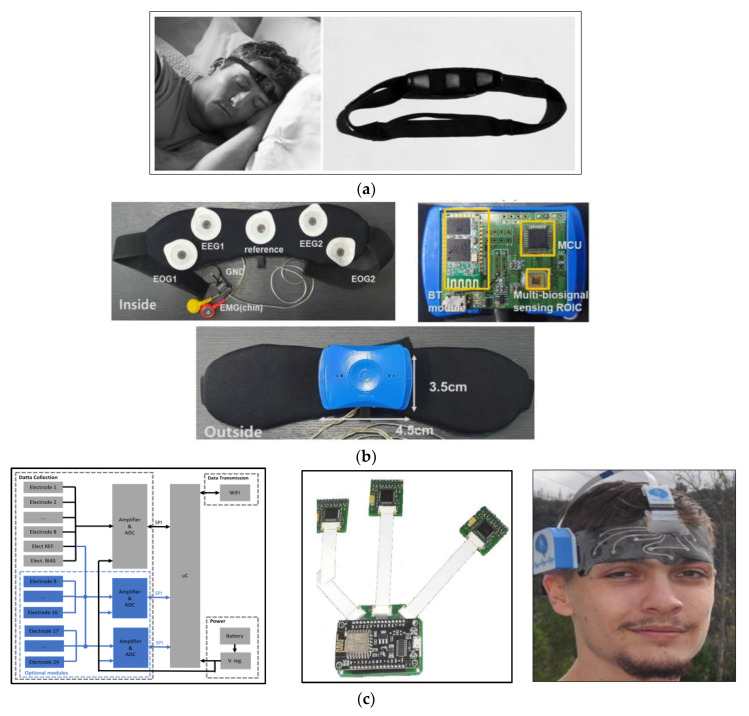
Flexible headbands. (**a**) The flexible headband with silver-coated fabric sensor [38,39]. (**b**) Components of the smart headband system: (upper left) the smart headband; (upper right) the sensor module; and (lower) the comprehensive multi-biosignal interface for sleep monitoring [40]. (**c**) The e-textile headband system overview: (left) system block diagram; (middle) processing and transmission board for the e-textile EEG system connected to multiple amplifiers; and (right) e-textile headband in use [41].

This system represents a marked departure from conventional sleep-monitoring methods to more user-friendly ones, as it is an adjustable headband designed to accommodate various individuals wearing it by having the right balance, i.e., tight enough to ensure security while loose enough to minimize discomfort. For a visual representation of the entire system, please refer to Figure 3a.

To validate this system, sleep data were collected from 29 healthy subjects using both PSG and the designed system in a sleep laboratory setting over the course of one night. The data were meticulously recorded and assessed by two proficient technicians. The outcomes of the present study strongly suggest that the designed system holds the potential to serve as a user-friendly and precise adjunct to existing, well-established technologies employed for sleep measurement in the context of healthy adults.

#### 2.2.2. Smart Headband

In 2019, Sung-Woo and his colleagues designed a wearable multi-biosignal wireless device for sleep analysis [40]. This system consists of a rubber and mesh headband, electrodes, and a sensor module box to acquire EEG, EMG, and EOG signals required for sleep analysis.

The electrodes employed are Ag/AgCl, strategically positioned following the 10–20 system, specifically at Fp1, Fp2, and Fpz, as indicated by EEG1, EEG2, and the reference in Figure 3b (upper left).

The sensor module box includes a multi-biosignal sensing ROIC, a low-power MCU, and a Bluetooth module. The headband dimensions are approximately 3 cm × 12 cm, while the module box is relatively compact, measuring about 3.5 cm × 4.5 cm in size, as shown in Figure 3b (upper right).

The ROIC is composed of two main sections: the analog front-end (AFE) section and the feature extraction section. The AFE encompasses a low-noise amplifier employing chopper stabilization to rectify DC offset, a programmable gain amplifier, and a low-pass filter. Its integration enhances the system’s compactness and efficiency while maintaining a small size and low power consumption.

The feature extraction and classification section take on the responsibility of sleep analysis through a rule-based decision tree integrated into the MCU, which uses the time-domain feature extraction data gathered by the analog circuit.

This system was designed to offer dual functionalities: wireless multi-biosignal monitoring and direct sleep-stage detection, validated by comparing it with the commercial OpenBCI system.

#### 2.2.3. E-Textile Headband

In 2020, Carneiro and his collaborators developed a wearable and comfortable e-textile headband designed for long-term forehead EEG signal acquisition [41]. Their aim was to address the challenges posed by the bulky size and complex wiring of current EEG monitoring systems, which often require significant time for set-up.

Their novel system is comprised of two main components: the skin interface e-textile and the electronic system. Firstly, the multi-layer stretchable e-textile was fabricated by integrating all electrodes into the headband, eliminating the need for individual electrode placement and intricate wiring. The fabrication process is extensively detailed in [41] in section (II) (A).

The schematic representation of the textile patch, along with its corresponding dimensions, including the precise placement of each printed electrode according to the international 10–20 EEG electrode positioning system, is vividly depicted in Figure 3c (upper). The electrodes are placed at (AF8, AF10, Fp10, Fp2, Fp1, Fp9, AF7, and AF9), yielding a total of 24 EEG channels. Secondly, the electronic system was designed to include two main components: an amplification circuit and a processing circuit. To capture the EEG signals, the system employs ADS1299, which adds a high degree of integration leading to enhanced system functionality, precise EEG signal acquisition, optimized power consumption, and efficient system operation. The overall system design is shown in Figure 3c (lower left).

This system was tested by recording EEG data during sleep, as shown in Figure 3c (lower right), and manually visualizing the differences in brain activity without doing any complex analysis.

It is important to mention that the person being tested felt fine during sleep and did not have any skin problems or irritation when they woke up.

Table 3 provides a comprehensive comparative analysis of the three flexible head- bands that were previously discussed. The e-textile headband boasts a greater number of channels compared to the smart headband, thereby enhancing its capacity to accurately capture EEG signals due to its high resolution. However, it is essential to note that the e-textile headband exclusively acquires EEG data, whereas the other two flexible headbands have an additional capability of capturing other physiological signals that enriches the dataset and provides more comprehensive insights into the subject’s sleep patterns, as elaborated in Table 1.

It is important to note that the smart headband is purposefully engineered for the task of sleep monitoring and staging. Conversely, the e-textile headband was limited to sleep monitoring and lacked comprehensive data analysis, relying solely on visual inspection. This makes the smart headband study more reliable, establishing it as a more dependable option to facilitate effective sleep monitoring and staging.

### 2.3. Highly Flexible EEG Sleep-Monitoring Systems

This section includes EEG sleep-monitoring systems with enhanced properties that make them more flexible than their predecessors. These systems prioritize user comfort during sleep while seamlessly integrating with the body to facilitate high-quality physiological monitoring. Nowadays, significant research endeavors are directed toward soft wearable systems to advance various aspects of epidermal electronics, particularly for diverse applications in healthcare [62,63].

In this section, we will delve into the realm of soft electrode-based EEG sleep-monitoring systems and introduce four cutting-edge systems designed for sleep monitoring: trEEGrid; a tattoo-based sleep-monitoring system; a soft electrode array-based REM sleep stage monitoring system; and at-home wireless sleep-monitoring patches.

#### 2.3.1. trEEGrid: Pre-Gelled Electrode Grid-Based Sleep-Monitoring System

In 2022, da Silva Souto and his colleagues conducted a remarkable study [42] in which they designed a pre-gelled electrode grid system for EEG sleep monitoring, eliminating the need for specialized personnel and enabling convenient use in a home setting. They called this innovative system the “trEEGrid.”

This system was inspired by an ear-EEG system called cEEGrid [57]—detailed later—and substantial modifications were applied to it to enhance the quality of sleep EEG data acquisition.

**Figure 4:**
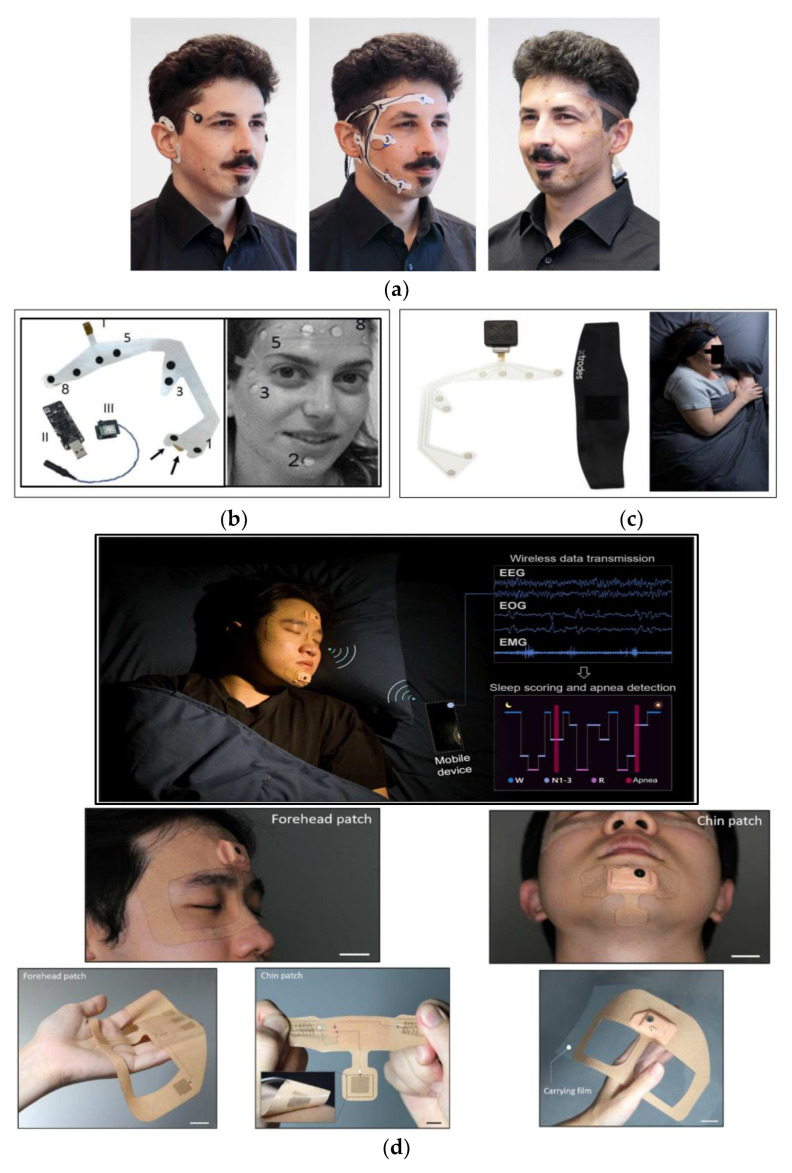
Highly flexible EEG sleep-monitoring systems. (**a**) Development steps of the self-applicable, pre-gelled trEEGrid: (left) step 1: integration of cEEGrid and EOG; (middle) step 2: foam trEEGrid prototype with ECG electrodes; and (right) step 3: trEEGrid prototype mounted on a flexible PCB [42]. (**b**) Tattoo-based monitoring system: (left) the electrode array system: (I) dry electrodes, (II) Bluetooth low-energy receiver, (III) amplifier and Bluetooth low-energy transmitter; and (right) system in use [24]. (**c**) Soft EEG, EOG, and EMG electrode array and the DAU [43]. (**d**) (upper) At-home sleep-monitoring patches for assessing sleep quality and sleep apnea; (middle) soft wearable patches conformally attached to the facial area; and (lower) forehead patch on a device carrier made of PTFE for convenient handling and storage for multiday use, showing backside of the highly flexible soft patch [44].

The development of the trEEGrid system can be delineated into three crucial steps: step 1, step 2, and step 3. Step 1 was based on the cEEGrid and EOG electrodes, forming the basis for their subsequent advancements, while step 2 focused on the creation of the system using pre-gelled neonatal ECG electrodes. These electrodes were meticulously integrated into a self-adhesive grid using medical foam plaster and played a pivotal role in validating and finalizing the trEEGrid design, constituting what they now refer to as step 3, which is considered a future development in their study. Figure 4a provides a visual representation of this progression.

The trEEGrid underwent validation through simultaneous recordings alongside a commercial PSG system known as the SOMNOscreen Plus. Data were collected from 32 subjects to compare the quality of EEG signals acquired by both systems. trEEGrid data underwent meticulous preprocessing, encompassing band-stop filtering, bandpass filtration, downsampling, and referencing. To annotate the data, a seasoned expert with 15 years of experience evaluated consecutive 30 s epochs of sleep data, and no feature extraction or classification algorithm was used. The results clearly demonstrate that the new array can be used effectively by healthy participants without the need for on-site hands-on assistance.

Additionally, this system was compared to standard PSG recordings, yielding a strong agreement indicated by a Cohen’s kappa coefficient of 0.70 ± 0.01. These findings strongly suggest that flexible, printed, pre-gelled sensor arrays designed for sleep EEG acquisition have the potential to enable convenient self-recording in a home environment.

#### 2.3.2. Tattoo-Based Sleep-Monitoring System

In 2019, Shustak and his colleagues designed a tattoo-based sleep-monitoring system [24] to overcome the limitations associated with traditional PSG methodologies, specifically the high costs, limited availability, and the labor-intensive nature of the procedure. Their innovation harnessed the potential of printed electrode technology, leading to the development of a soft skin-adhesive with no gel-based electrode home monitoring system for EEG, EOG, and EMG signals in the form of a tattoo-based solution.

The electrode array was designed by incorporating a variety of materials such as polyurethane films, silver, and carbon. This fusion resulted in an electrode array comprising four EEG electrodes for the forehead, two EOG electrodes, and two surface EMG electrodes. The integration of these components was complemented by a compact wireless recording system. This system effectively amplified and transmitted the collected data to a personal computer for subsequent post-processing. The system is shown in Figure 4b.

As a result, this tattoo-based wireless system effectively recorded EMG, EOG, and EEG signals. Consistent recordings were obtained in both hospital and home settings, and a 6 h sleep-monitoring session exhibited distinct differentiation of sleep stages, which was scored by a trained sleep technician according to the AASM manual.

This system proved its ability to carry substantial significance for monitoring sleep disorders within the domestic environment and to identify the conditions linked to neurological disorders including REM sleep behavior disorder.

#### 2.3.3. Soft Electrode Array-Based REM Sleep Stage Monitoring System

In 2023, Oz and his colleagues employed the same electrode array referenced earlier [24], with additional details available in references [64,65]. Their objective was to develop an at-home wearable system for sleep monitoring and to investigate its feasibility and validity compared to PSG [43].

The system includes soft, printed dry electrode arrays and a compact wearable data acquisition unit (DAU) securely fastened to the head using a headband. The DAU is managed through an Android application, and the collected data are initially stored on an integrated SD card and later transferred to the cloud for in-depth analysis. The headband, DAU, and electrode array can be observed in Figure 4c.

To validate the system, 50 elderly participants (21 healthy, 29 with Parkinson’s disease) underwent sleep recording from both PSG and this wearable system. Then, the two recordings were compared in terms of sleep staging, which was conducted manually by a sleep specialist, resulting in a strong agreement between them, with a Cohen’s kappa coefficient of 0.688. Consistent agreement was found across various sleep stages, including wakefulness (0.701) and REM sleep (0.723). Notably, the system demonstrated an 85.7% sensitivity in detecting REM sleep without atonia, and reduced awakenings during home sleep were noticed compared to controlled lab sleep. These results affirm the system’s validity, precision, and potential for widespread use in detecting sleep disorders, particularly in home-based settings, offering improved healthcare prospects.

#### 2.3.4. At-Home Wireless Sleep-Monitoring Patches

In 2023, Kwon and his research team [44] pioneered the design of an entirely wearable and exceptionally comfortable at-home sleep-monitoring system designed to assess both sleep quality and sleep apnea. This wearable system includes two compact patches: one for capturing EEG and EOG signals from the forehead, and the other for monitoring EMG activity in the chin area. These crucial physiological signals are continuously analyzed in real time to identify various sleep stages and detect potential sleep disorders.

This unobtrusive and soft patch has been set apart due to its smaller form factor compared to other wearable sleep monitors, as illustrated in Figure 4d. Its compact design allows for seamless integration with the skin, ensuring high fidelity and reliable signal detection throughout the entire sleep cycle.

EEG, EOG, and EMG are wirelessly collected and transmitted via Bluetooth to a mobile device, such as a smartphone or tablet, for further analysis. A convolutional neural network (CNN) algorithm is employed to automatically score sleep patterns in real time and to identify apnea events.

Figure 4d (middle) emphasizes the intimate contact between the wearable patch and the user’s face, specifically the forehead and chin regions. Figure 4d (lower) displays the front side of the soft membrane patch, conveniently mounted on a polytetrafluoroethylene (PTFE) substrate for easy handling. On the back side of the device, skin-contact nanomembrane electrodes are incorporated, boasting exceptional stretchability and flexibility.

This soft wearable platform prioritizes user comfort, ease of use, and portability. Users can effortlessly follow instructions to monitor their sleep in their homes without the need for the presence of technicians. In a clinical study, these face-mounted patches exhibited performance comparable to the gold standard of PSG.

As a groundbreaking achievement, this wearable system demonstrates an impressive (88.5%) accuracy in the detection of obstructive sleep apnea when comparing healthy control subjects with sleep apnea patients. Moreover, the integration of deep learning techniques facilitates automated sleep scoring, resulting in cementing the system’s portability and point-of-care usability.

Table 4 offers a concise comparison of the four EEG sleep-monitoring devices outlined in this section, focusing on fabrication material, as well as the number and type of EEG electrodes. Despite the fact that the device in [44] has fewer EEG channels compared to [24,42,43], the choice of fabrication material renders it highly adherent to the subject’s skin, enhancing overall comfort during use as well as EEG signal quality.

### 2.4. Ear-EEG Sleep-Monitoring Plugs and Patches

The idea behind the ear-EEG technology originates from the requirement for a discreet, unobtrusive, robust, user-friendly, and feasible EEG system for sleep monitoring [66]. The ear-EEG signal is captured through the integration of electrodes within a specialized earpiece. The electrode composition, amplification mechanisms, and underlying principles mirror those utilized in on-scalp EEG recordings. However, these systems have a reduced number of electrodes compared to the conventional EEG systems, but their efficacy in delivering high-quality EEG signals has been proven, especially in brain–computer interface applications [66,67,68]. Moreover, this technology has been recently used to monitor various physiological responses beyond EEG, including cardiac activity [69,70].

The ear-EEG wearable system is designed for long-term comfort, and its electrodes are securely placed inside the ear canal to ensure recordings of high-quality signals. Despite the low signal amplitude in comparison to scalp EEG, the signal-to-noise ratio (SNR) was found to be similar, highlighting its reliability [45,66,67].

It is important to note that the recorded EEG signals are prone to attenuation caused by various factors, such as the presence of cerebrospinal fluid, the properties of the skin, and the cranial bone. These attenuations are akin to those encountered in conventional scalp EEG systems.

#### 2.4.1. In-Ear Viscoelastic Earpiece

**Figure 5:**
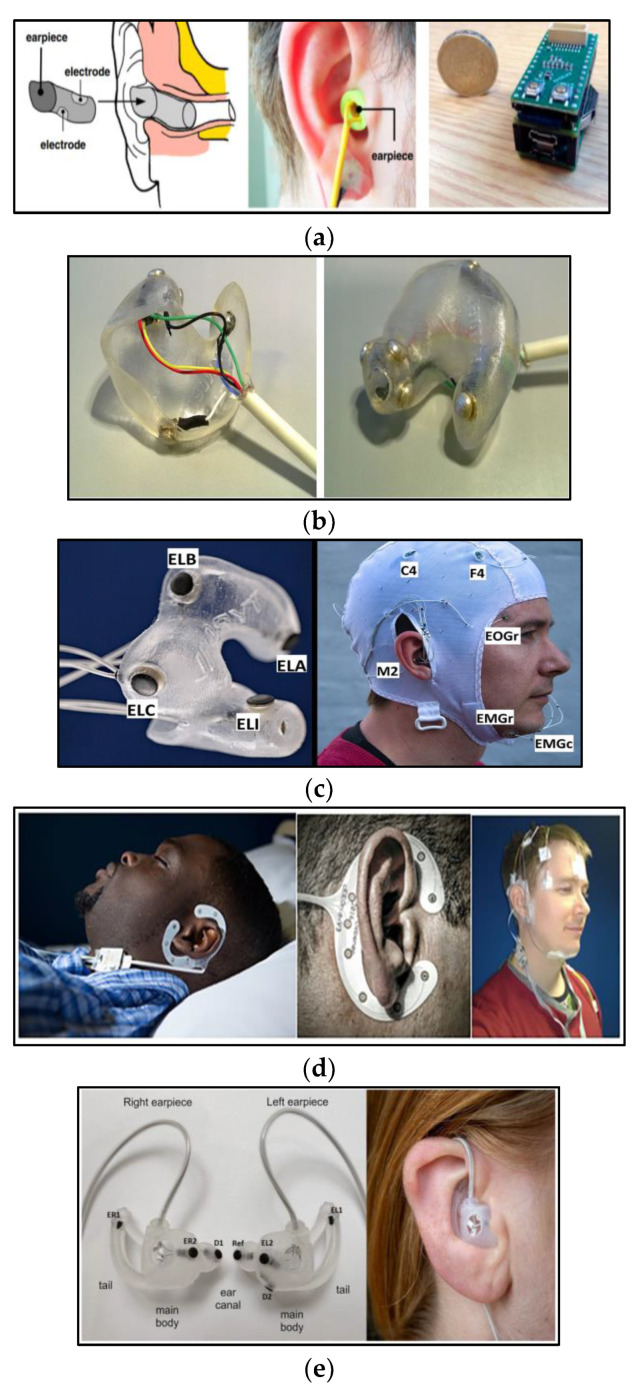
Ear-EEG for sleep monitoring. (**a**) In-ear viscoelastic earpiece: (left) the earpiece and its electrode positioning; (middle) earpiece in use; and (right) electronics platform [46]. (**b**) Wet electrode-based in-ear hardshell earpiece [52]. (**c**) Dry electrode-based in-ear hardshell earpiece [54]. (**d**) cEEGrid data collection set-up: (left) cEEGrid system set-up during sleep; (middle) close-up of cEEGrid; and (right) simultaneous montage of standard PSG and cEEGrid set-up [56]. (**e**) Generic earpieces: (left) earpiece’s electrode position (EL1, EL2, ER1, and Er2: data electrodes, D1 and D2 ground electrodes, ref: reference electrode); and (right) earpiece in use [58].

For a while, scalp EEG monitoring stood alone as the primary measurable indicator of neural activity during sleep. However, a remarkable shift occurred in 2016 when Looney and his team embarked on an investigation to assess the correlation between ear-EEG and scalp EEG [46]. Their study aimed to address the limitations associated with scalp EEG, specifically the high cost and the challenges it posed to patient comfort during sleep. At that time, they employed a recently developed ear-EEG system [45], designed by Goverdovsky and his colleagues. This system marked a departure from traditional silicone earmolds by offering a sensor based on a viscoelastic substrate and conductive cloth electrodes that demonstrated several favorable mechanical and electrical properties. The in-ear viscoelastic earpiece is shown in Figure 5a.

The viscoelastic property of this earpiece ensures that it snugly conforms to the contours of the ear canal, resulting in a stable electrode–skin interface. Additionally, the cloth electrodes require nothing more than a saline solution to establish a low-impedance connection with the skin.

The functionality of the system was confirmed by simultaneously acquiring sleep data from four healthy men during naps using the ear-EEG system and the conventional PSG. The collected data were meticulously analyzed by clinical experts, and a significant agreement between the recordings was obtained.

Moreover, these four healthy subjects’ data played a pivotal role in a study conducted by Nakamura and his colleagues [47]. They applied several preprocessing steps to enhance the quality of these data, including downsampling, exclusion of high-amplitude epochs, and bandpass filtering. After that, 30 features were extracted from the data, which include frequency domain features and structural complexity features. These features were then utilized as inputs for a one-against-one multi-class support vector machine (SVM) with a radial basis function (RBF) kernel serving as the classifier. This work showcased the feasibility of utilizing ear-EEG for out-of-clinic sleep monitoring, particularly in the context of automatic sleep stage classification.

In 2018, Alqurashi and his colleagues conducted a comparative study [48] using the same system. They measured ear-EEG alongside commercial PSG data from 21 subjects, demonstrating the system’s efficacy in detecting slow-wave sleep (SWS), measuring sleep latency, and automating the five-stage sleep scoring process. The automated scoring achieved with in-ear EEG yielded a Cohen’s kappa coefficient of 0.61 compared to manual scoring, and 0.79 compared to scalp EEG scoring. This suggests that the in-ear EEG system exhibited notable proficiency in sleep stage classification, approaching the accuracy achieved by traditional scalp EEG methods.

Additionally, Nakamura and his research team relied on the same dataset [49] to explore the potential of ear-EEG in the automatic detection of drowsiness, specifically, distinguishing between wakefulness and light sleep. In 2020, the same group utilized the same system to investigate the potential of ear-EEG technology for overnight sleep monitoring [50]. Their study involved 21 subjects whose sleep patterns were simultaneously monitored using both PSG and the ear-EEG system. The acquired data were analyzed for both structural complexity and spectral domains, and their findings suggest that the in-ear sensor is a viable option for monitoring overnight sleep beyond the confines of a sleep laboratory. Moreover, it effectively addresses the technical challenges often associated with PSG, making it a compelling 24/7 wearable alternative to conventional, cumbersome, and costly sleep-monitoring equipment.

#### 2.4.2. Wet Electrode-Based In-Ear Hardshell Earpiece

In 2017, Mikkelsen and his team introduced an in-ear EEG sleep-monitoring system [52] to measure cerebral activity and to automatically classify sleep into five stages. They employed a device that had been developed by their team in 2015 [51], which consists of a customized hardshell in-ear EEG crafted from soft silicon material. This device includes six electrodes integrated into the same earpiece and referenced to a passive electrode positioned at Cz. These electrodes are solid silver buttons connected to copper wires, as depicted in Figure 5b.

Sleep data were simultaneously collected using the ear-EEG system and conventional PSG, and data from both systems were compared. Many features were extracted from the ear-EEG system data, including signal skewness, signal kurtosis, and Hjorth complexity. Subsequently, a random forest algorithm was utilized as the classifier.

This study’s findings indicate that ear-EEG recordings contain valuable information pertaining to sleep stages. They also reveal that automated sleep staging using ear-EEG technology can accurately classify sleep stages, making it a pertinent tool for both scientific research and clinical sleep assessments. Furthermore, the advantages of ear-EEG-based scoring become evident when compared to PSG in terms of its high mobility and cost effectiveness.

In a separate investigation [53], the same ear-EEG device was utilized to explore the potential applicability of its recording in monitoring overnight sleep EEG activity. Sleep data were collected from a single participant, and they verified the congruence of both temporal and spectral characteristics between ear-EEG and traditional scalp EEG recordings.

#### 2.4.3. Dry Electrode-Based In-Ear Hardshell Earpiece

In 2019, again Mikkelsen and his team employed an alternative type of ear-mounted EEG measurement system with 12 dry electrodes distributed among two earplugs [54], as shown in Figure 5c.

This system was evaluated by conducting 80 sleep recordings of 20 subjects, using the ear-EEG system and conventional PSG. Data acquired by the ear-EEG system underwent preprocessing, involving notch filtering, spike rejection, and electrode rejection. Following this, each recording was divided into non-overlapping 30 s epochs. Subsequently, approximately 83 features, including signal skewness, signal kurtosis, Hjorth complexity, and others, were extracted. To classify the data, a random forest algorithm was employed as the classifier.

The results were quite promising, as the system demonstrated high levels of convenience and comfort, and 19 out of 20 subjects reported minimal or no adverse effects on their sleep; they found it easy to wear the system without supervision during their sleep sessions.

Furthermore, this team implemented a machine learning approach trained with the sleep EEG data collected from the system that led to the development of an automated scoring algorithm, which achieved an impressive Cohen’s kappa value of 0.73 for five-stage sleep scoring compared to manual scoring based on PSG data.

#### 2.4.4. cEEGrid: Flex-Printed Ear-EEG

The cEEGrid was crafted in 2015 by Debener and his collaborators [55] using 10 reusable, flexible, printed Ag/AgCl electrodes arranged in a C-shaped configuration snugly fitted around the ear.

This system was evaluated by Sterr and his team [56]. Their aim was to determine the suitability of cEEGrid for sleep research and assess its signal quality for sleep stage scoring. They collected sleep data using both the cEEGrid and PSG from 20 participants in a sleep laboratory, as illustrated in Figure 5d. Then, they highlighted the convenience of the cEEGrid set-up for sleep monitoring and demonstrated the user’s ability to independently complete the measurement set-up in just 20 min, compared to 45 min for set-up and trained sleep technician assistance required in its PSG counterpart.

To establish the reliability of the cEEGrid system, the collected data were manually scored by trained experts, and the results show an average Cohen’s kappa coefficient of about 0.42 indicating the agreement between the two systems’ scores.

In 2021, da Silva Souto and his research team conducted a comprehensive investigation of the efficacy of flexible printed electrodes for sleep monitoring in a smartphone-based home environment [57]. While the previous study in [56] assessed the cEEGrid’s performance solely within a laboratory setting for one night’s sleep, this study took a novel approach by evaluating sleep at home for ten participants who were equipped with the cEEGrid and a portable amplifier. The EEG data were recorded for 7.48 h from Fpz, EOG_L, and EOG_R positions and wirelessly streamed to a smartphone.

Hypnograms were generated using a sophisticated sleep scorer by analyzing EEG data from those positions and used as a reference baseline for comparison. Moreover, the scorer created alternative hypnograms by employing diverse combinations of cEEGrid channels and EOG channels. The compelling findings emerging from the comparison of hypnograms rooted in frontal electrodes with those based on cEEGrid electrodes emphasized the ear-EEG system’s ability to accurately identify distinct sleep stages. This re-markable outcome holds promising implications hinting at the development of compact, user-friendly ear-EEG systems that individuals can comfortably employ at home.

#### 2.4.5. Generic Ear-EEG

In 2023, Tabar and his team successfully developed a versatile ear-EEG system comprised of two generic earpieces equipped with embedded dry electrodes. Their primary objective was to offer ear-EEG-based sleep monitoring to a broad spectrum of the population without customizing the device to individual users [58]. They designed a generic earpiece with integrated dry electrodes to ensure user comfort and good contact between the electrodes and the body during sleep.

To achieve these goals, they precisely crafted four distinct earpieces tailored to the variations in the anatomical shapes and sizes of the human ear. These earpieces maintained a consistent shape while varying in size to suit different individuals. These earpieces were expertly molded from a soft silicone material, endowing the system with the mechanical properties required for optimal performance. Each of these earpieces was equipped with two electrodes, as illustrated in Figure 5e.

This system can automatically score sleeping in three steps: preprocessing, feature extraction, and classification. The preprocessing step involves bandpass filtering, notch filtering, and removal of artifacts and high-amplitude spikes, followed by dividing the ear-EEG recordings into non-overlapping 30 s epochs. The feature extraction step is the step where 84 features are extracted, including time domain features, frequency domain features, and others. Then, the epochs can be classified using a five-class random forest classifier consisting of 100 decision trees.

The ear-EEG system was validated by comparing its automated sleep scoring with PSG-based scoring, revealing a substantial level of agreement with a kappa value of 0.71. Also, a comparison was made between the designed earpiece and customized ones, concluding that the type of earpiece used does not affect the measured data. These findings underscore the potential of a generic ear-EEG as a promising alternative to PSG.

Table 5 summarizes the systems discussed in this section, providing a comparison in terms of ear-EEG type, fabrication material, electrode type, and the associated sleep assessment studies for each system.

## 3. In-Depth Analysis among the Categorized Wearable EEG-Based Sleep Monitoring Systems

After thoroughly addressing and discussing various types of wearable EEG devices employed for sleep monitoring, it is essential to present a comparison among them in terms of system material, EEG electrode material/type, system performance, system wearability, usability, and obtrusiveness, and the system’s targeted age group.

Table 6 provides a summary of the materials employed for each reported technique, accompanied by a thorough exploration of their respective performance matrices. While several studies did not delve into the details about the materials used in designing their sleep-monitoring devices, others provided insights through relevant tests, shedding light on the advantages associated with their chosen materials.

In [41], the mechanical characteristics of the e-textile headband were meticulously explored, with a focus on its primary components—latex and Lycra. The assessment involved subjecting the headband to stretching, accompanied by a simultaneous evaluation of its electrical resistance.

At 20% stretch, the electrical resistance doubled. However, this increase is relatively insignificant given the initially low resistance. In practical scenarios, such as wearing the headband, it is unlikely to stretch beyond 20%.

At 32% stretch, a sudden surge in electrical resistance was noted. This indicates that stretching beyond 32% is excessive for the headband, suggesting a limit for optimal usage.

In [44], the characteristics of the materials employed in their system were thoroughly investigated. Their analysis included a stretchability assessment, revealing that even after 1000 cycles of 30% stretching, minimal changes in resistance occurred. This result underscores the device’s enduring reliability for repeated usage. Notably, the wearable device’s fabric exhibited remarkable stretchability, surpassing 300%, and retained its elasticity through numerous stretching cycles, affirming its suitability for prolonged wear.

The examination extended to the soft silicone adhesive material utilized in the device, focusing on thickness and peeling strength. The researchers determined that a 250-μm-thick membrane struck the optimal balance, providing sufficient peeling strength while ensuring conformity to the skin.

Furthermore, the study delved into the device’s performance during long-term wear and its cleaning requirements. Over a continuous seven-day period, the device demonstrated consistent performance, attesting to its durability. Notably, washing the device with soap emerged as an effective method to maintain its peeling strength. This finding underscores the device’s capability for prolonged and reliable use, aligning with the demands of real-world applications.

**Figure 6:**
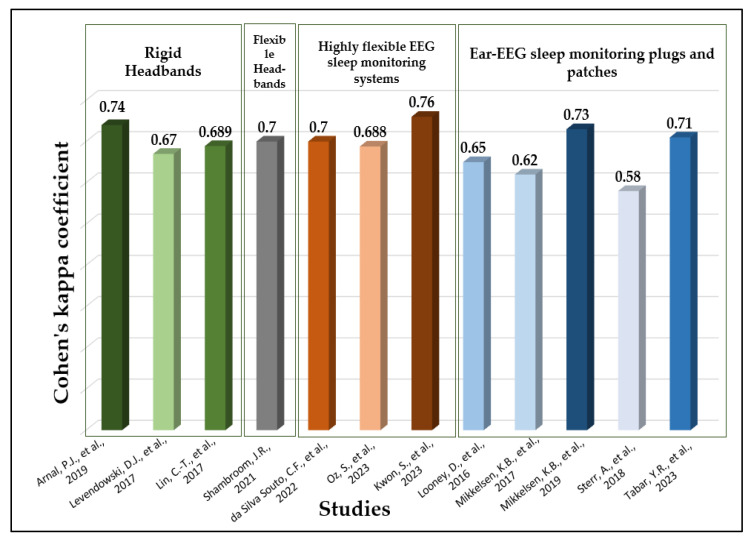
Cohen’s kappa coefficient for EEG-based sleep-monitoring systems compared to PSG [29,36,38,39,42,43,44,46,52,54,56,58].

In terms of the performance matrix, most studies attempted to measure Cohen’s kappa coefficient reflecting the system agreement with the PSG. Figure 6 and the last column of Table 6 provide the Cohen’s kappa coefficient of each system compared to PSG. The wireless sleep-monitoring patch stated in [44] demonstrated the highest coefficient among the reported technologies with a significant value of 0.76. This emphasizes the robustness and reliability of this system in accurately classifying sleep stages compared to PSG.

Addressing the degree of wearability/usability/obtrusiveness for each of the EEG-based sleep-monitoring systems is important. It is worth noting that none of the systems discussed employs any metrics for assessing wearability, usability, and obtrusiveness, except for the study [54,58], which employed a sleep questionnaire for user experience evaluation, and [41,44], which concentrated on the mechanical characteristics of the underlying system for the wearability assessment.

In our evaluation of the wearability, usability, and obtrusiveness of each device, we relied on our own understanding. We assessed the device’s wearability by considering factors such as comfort, ease of application and removal, adjustability, and battery life. Simultaneously, we evaluated usability in terms of user interface, training, and system setup.

**Figure 7:**
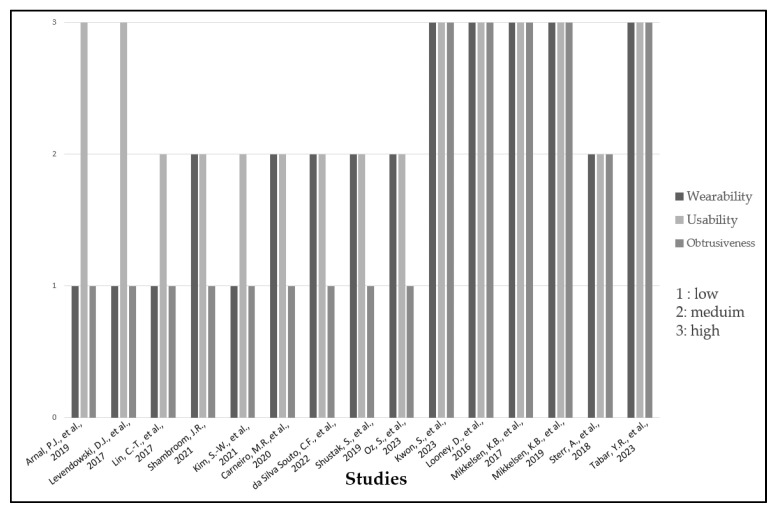
EEG-based sleep-monitoring systems’ wearability, usability, and obtrusiveness [24,29,36,38,39,40,41,42,43,44,46,52,54,56,58].

To assess the system’s obtrusiveness, our assessment included factors like device size, bulkiness, weight, and visibility to others. We utilized a methodology that assigns levels (high, medium, or low) to each device, as illustrated in Figure 7.

Upon careful examination of Figure 7, it is evident that the ear-EEG sleep-monitoring systems and the patch discussed in [44] exhibit the highest degrees of wearability, usability, and obtrusiveness compared to other systems. It is essential to highlight that even though these systems exhibit the highest degrees of wearability, usability, and obtrusiveness, the limitations—outlined in the outlook and summary section—should be considered. It is important to note that this representation is purely comparative in nature.

Lastly, Table 7 summarizes participants’ information across all studies mentioned above. This table encompasses details such as participant numbers, ages, and categories, thereby offering a thorough overview and clear understanding of the targeted age groups for each system.

It is essential to emphasize that although the reviewed studies did not explicitly specify their targeted age groups, we meticulously extracted data from participant information to deduce the intended age demographic. As illustrated in Table 7, most of the studies were geared towards adults. However, we posit that many of these systems are versatile and can cater to both adult and elderly populations. Notably, study [43] stood out as the lone research endeavor explicitly focusing on the elderly demographic.

## 4. Summary and Outlooks

Sleep is considered one of the most vital natural processes necessary for physical and mental health. Our brain requires sufficient and high-quality sleep to operate effectively; as a result, we can focus, think clearly, and retain a solid memory while reducing the risk of various neurodegenerative diseases. For these reasons, the concept of sleep monitoring has seen an upward trend among scientific researchers, leading to the development of different sleep-monitoring systems with different designs.

PSG has historically stood as the benchmark for assessing sleep, but its limitations have spurred the evolution of wearable home-based monitoring systems that have emerged as a promising solution, offering more convenient, comfortable, and cost-effective systems for tracking sleep quality and patterns.

Unlike previously published review papers, this study intended to delve deeply into the crucial domain of sleep monitoring, highlighting different wearable sleep-monitoring systems reported in the scientific literature, especially systems that collect and process EEG signals that function as the principal indicator for distinguishing between different sleep stages and identifying sleep disorders.

We divided wearable EEG-based sleep-monitoring systems into four main categories, namely, rigid headbands, flexible headbands, highly flexible EEG sleep-monitoring systems, and ear-EEG sleep-monitoring plugs and patches. Within each category, we dig deeply to explore many systems in terms of their design, form factor, materials, and methods of sleep assessment, and a comprehensive comparative table summarizing the key features of each system is sincerely conducted.

Historically, rigid headbands have served as the backbone of wearable EEG-based sleep-monitoring systems, exemplified by models like the Dreem Headband and the Sleep Profiler. These systems use electrodes with different types and fabrication materials to collect EEG signals from specific positions with enough resolution. However, it has a limitation of being uncomfortable for the user during sleep, as it is manufactured from rigid-state materials, leading to the need for developing more user-friendly alternatives such as flexible headbands.

Flexible headbands are designed to conform to the contour of the head when worn and to ensure secure attachment to the skin. They are fabricated from many materials, such as textiles and rubber. Furthermore, highly flexible sleep-monitoring systems enhance user comfort during long-term monitoring and precise detection of neurological disorders. One of these systems is the soft technology-based EEG sleep-monitoring patch, which took comfort and wearability to the next level by introducing soft skin-integrated electrode arrays that seamlessly conform to the user’s body. Patches developed by researchers like Kwon and his team prioritize the requirements for user comfort and the system’s easy use while demonstrating impressive accuracy in detecting sleep disorders, including obstructive sleep apnea.

As future research trends toward soft patches, numerous factors must be considered to enhance the breathability and reusability of wearable electrodes. The development of a replaceable adhesive layer for the electrode not only reduces costs but also facilitates the long-term utilization of wearable sleep monitors. Moreover, the integration of additional sleep sensors to measure parameters such as blood oxygen saturation, carbon dioxide levels, and motion provides a richer set of sleep data that can complement EEG measurements. This holistic approach to monitor various sleep-related factors ensures a more comprehensive understanding of sleep patterns and contributes to the advancement of sleep research.

Another striking development is the emergence of ear-EEG sleep-monitoring systems, marking a significant shift in sleep-monitoring technology. These systems combine comfort and wearability with discreet in-ear sensing technology. Despite having fewer electrodes than traditional EEG systems, ear-EEG systems have proven their reliability and a high signal-to-noise ratio. They are particularly well suited for brain–computer interfaces and have shown efficacy in sleep stage classification. Ear-EEG systems, such as in-ear viscoelastic earpieces, hardshell earpieces with wet electrodes, and generic earpieces with embedded dry electrodes, offer promising alternatives to traditional PSG methods.

Despite the outstanding advancements in ear-EEG technology, it still grapples with certain challenges and limitations. The primary drawback lies in its constrained coverage area, leading to a diminished collection of sleep-related data. Addressing this limitation requires a strategic shift in future research efforts, emphasizing signal processing and machine learning methodologies. Specifically, there is a need for signal estimation techniques that can accurately deduce EEG signals from a targeted brain area using exclusively the data obtained from an ear-EEG. This targeted approach holds the potential to significantly augment the quality of ear-EEG signals [70].

By shedding light on wearable EEG sleep-monitoring systems, this review paper enriches our understanding of sleep quality and overall well-being while providing a good reference to designers to overcome the limitations of the most recent EEG sleep-monitoring systems.

## Figures and Tables

**Table 1:** Overview of recently developed wearable EEG systems for sleep monitoring.

No.	Category	Systems	Data	Related Studies	Year
1	Rigid headbands	The Dreem Headband (DH).	EEG, movement, position, breathing, and heart rate	[29]	2019
				[30]	2021
				[31]	2022
				[32]	2023
				[33]	2022
		2. The Sleep Profiler (SP).	EEG, pulse rate, head movement and position	[34]	2016
				[35]	2016
				[36]	2017
				[37]	2023
		3. Rigid headband with flexible EEG dry sensor.	Only EEG	[38]	2017
2	Flexible headbands	Flexible headband with silver- coated fabric dry sensor.	EEG and signals from eye movements and the frontalis muscle	[39]	2012
		2. Smart headband.	EEG, EMG, and EOG	[40]	2020
		3. E-textile headband.	Only EEG	[41]	2020
3	Highly flexible EEG sleep-monitoring systems	trEEGrid: pre-gelled electrode grid-based system.	EEG, EMG, and EOG	[42]	2022
		2. Tattoo-based monitoring system.	EEG, EMG, and EOG	[24]	2019
		3. Soft electrode array-based REM sleep stage monitoring system.	EEG, EMG, and EOG	[43]	2023
		4. At-home wireless sleep-monitoring patches.	EEG, EMG, and EOG	[44]	2023
4	Ear-EEG sleep-monitoring plugs and patches	In-ear viscoelastic earpiece.	EEG	[45]	2015
				[46]	2016
				[47]	2017
				[48]	2018
				[49]	2018
				[50]	2019
		2. Wet electrode-based in-ear hardshell earpiece.	EEG	[51]	2015
				[52]	2107
				[53]	2016
		3. Dry electrode-based in-ear hardshell earpiece.	EEG	[54]	2019
		4. cEEGrid: flex-printed ear-EEG.	EEG	[55]	2015
				[56]	2018
				[57]	2021
		5. Generic ear-EEG.	EEG	[58]	2023

**Table 2:** Feature comparison among the three rigid headbands.

No.		DH [29]	X4 SP [36]	Forehead Headband [38]
1	Year of the study	2019	2017	2017
2	Fabrication process	-	-	Discrete
3	Number of EEG channels	5	3	4
4	EEG electrode position	F7, F8, Fpz, O1 and O2	AF7, AF8, and Fpz	AF5, Fp1, Fp2, and AF8
5	EEG electrode type	Dry EEG electrodes	Conventional gel (wet)	Dry EEG electrodes
6	EEG electrode material	Silicon	Ag/AgCl	Silicon-based
7	EEG resolution (bit)	-	-	24
8	EEG sampling rate (SPS)	256	256	250/500
9	Power	Rechargeable battery	Rechargeable battery	Rechargeable battery (750 mAh, 3.0 V) Li-ion battery
10	Power consumption	-	-	225 mW
11	Recording time	more than 10 h	up to 16 h	8 to 10 h
12	Size of control unit	-	-	30 × 25 × 5 mm^3^
13	Weight	-	2.5 oz	-

**Table 3:** Feature comparison among flexible headbands.

No.		[34]	[40]	[41]
1	Year of the study	2012	2019	2020
2	Fabrication process	Discrete	ASIC	Discrete
3	Number of EEG channels	1	2	24
4	EEG electrode position	Fp1 and Fp2	Fp1, Fp2, and Fpz	AF8, AF10, Fp10, Fp2, Fp1, Fp9, AF7, and AF9
5	EEG electrode type	Dry EEG electrodes	Dry EEG electrodes	Dry EEG electrodes
6	EEG electrode material	Silver-coated fabric	Printed composite	Textile printed
7	EEG resolution (bit)	12	16	24
8	EEG sampling rate (SPS)	128	-	250
9	Power	-	Rechargeable battery	two (7.4 V, 1600 mAh) LiPo cells
10	Power consumption	-	70.9 mW	-
11	Recording time	-	-	24 h
12	Size of control unit	-	3.5 cm × 4.5 cm	-

**Table 4:** Feature comparison among the highly flexible EEG sleep-monitoring systems.

Study	Year	Fabrication Material	Number of EEG Electrodes	EEG Electrode Type
[42]	2022	-	4	Pre-gelled neonatal ECG electrodes
[24,43]	2019, 2023	Polyurethane films, silver, and carbon	4	Soft, printed dry electrode arrays
[44]	2023	Metals, polymers, and silicon	2	Dry electrode

**Table 5:** Feature comparison among ear-EEG plugs and patches and their related sleep assessment studies.

Study	System	Ear-EEG Type	Fabrication Material	Number of Electrodes	EEG Electrode Type	Sleep Assessment Study
[45]	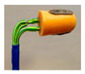	In-ear viscoelastic earpiece	Memory foam substrate	2	Cloth electrodes	[46] Comparison of manually scored hypnograms based on ear-EEG and scalp EEG. [47] Comparison of automatic sleep scoring based on ear-EEG and scalp EEG. [48] Comparison of sleep latency based on ear-EEG and scalp EEG. [49] Automatic detection of drowsiness based on ear-EEG. [50] Automatic overnight sleep staging using ear-EEG.
[51]	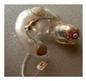	In-ear hardshell earpiece	Soft silicon material	6	Solid silver wet electrodes	[52] Automatic overnight sleep staging using ear-EEG. [53] Comparison of ear-EEG with standard scalp EEG visually and using power spectrograms.
[54]	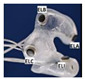	In-ear hardshell earpiece	Soft silicon material	6	Iridium-oxide dry electrodes	[54] Comparison of automatic sleep scoring based on ear-EEG and scalp EEG.
[55]	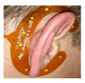	C-shape around-ear	-	10	Flexible printed wet Ag/AgCl electrodes	[56] Assessing the feasibility of cEEGrid for sleep stage scoring, a comparative study with standard PSG. [57] Comparative analysis of hypnograms, manual scoring of sleep stages using ear-EEG and scalp EEG.
[58]	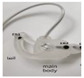	In-ear hardshell earpiece	Soft silicon material	2	Dry electrodes	[58] Comparison of automatic sleep scoring based on ear-EEG with PSG-based sleep scoring performed by a professionally trained sleep scorer.

**Table 6:** Comparison among EEG-based sleep-monitoring systems in terms of system material, EEG electrode material/type, and system performance.

No.	Category	System	System Material ^1^	EEG Electrode Type /Material ^2^	System Performance (Cohen’s Kappa Coefficient)
1	Rigid headbands	The Dreem Headband (DH)	Foam, fabric, and TPU	Silicone	0.74
		2. The Sleep Profiler (SP).	-	Ag/AgCl	0.67
		3. Rigid headband with flexible EEG dry sensor.	-	Silicon	0.689
2	Flexible headbands	Flexible headband with silver-coated fabric dry sensor.	Plastic	Silver-coated fabric	0.7
		2. Smart headband.	Rubber and mesh material,	Printed composite	-
		3. E-textile headband.	Textile printed	Textile printed (Lycra-mesh fabric, latex, and others)	-
3	Highly flexible EEG sleep-monitoring systems	trEEGrid: pre-gelled electrode grid-based system.	Medical foam plaster (does not specifies the material)	Pre-gelled neonatal ECG electrodes (does not specifies the material)	0.7
		2. Tattoo-based monitoring system.	Polyurethane films, silver, and carbon	Printed dry electrode	-
		3. Soft electrode array-based REM sleep stage monitoring system.	Polyurethane films, silver, and carbon	Printed dry electrode	0.688
		4. At-home wireless sleep monitoring patches.	Metals, polymers, and Silicon	-	0.76
4	Ear-EEG sleep-monitoring plugs and patches	In-ear viscoelastic earpiece.	Memory foam substrate	Cloth electrodes	0.65
		2. Wet electrode-based in-ear hardshell earpiece.	Soft silicon material	Solid silver wet electrodes	0.62
		3. Dry electrode-based in-ear hardshell earpiece.	Soft silicon material	Iridium-oxide dry electrodes	0.73
		4. cEEGrid: flex-printed ear-EEG.	-	Flexible printed wet Ag/AgCl electrodes	0.58
		5. Generic ear-EEG.	Soft silicon material	Dry electrodes	0.71

^1^ For all systems, no side effects on human skin were reported when using them. ^2^ For some systems, the ‘’EEG electrode material” is replaced with “EEG electrode type” when the system’s study does not specify the material directly, facilitating meaningful comparisons across all systems.

**Table 7:** Participant information from the studies related to EEG-based sleep-monitoring devices.

No.	Category	System	Related Studies	Participant Number	Participant Age (Years)	Participant Category
1	Rigid headbands	1. The Dreem Headband (DH).	[29]	31	18–65	Adults + Elderly
			[30]	100	60 and older	Elderly
			[31]	21	18 and above	Adults + Elderly
			[32]	25	17–23	Adults
			[60]	21	29 ± 5	Adults
		2. The Sleep Profiler (SP).	[34]	14	22–34	Adults
			[35]	29	25–80	Adults + Elderly
			[36]	47	60.7 ± 14.7	Elderly
			[37]	26	64.6 + 13.0 and 63.2 + 12.7 (two sites)	Elderly
		3. Rigid headband with flexible EEG dry sensor.	[38]	10	24 ± 6	Adults
2	Flexible headbands	1. Flexible headband with silver- coated fabric dry sensor.	[39]	29	19–60	Adults + Elderly
		2. Smart headband.	[40]	-	-	-
		3. E-textile headband.	[41]	-	-	-
3	Highly flexible EEG sleep-monitoring systems	1. trEEGrid: pre-gelled electrode grid-based system.	[42]	12	18–45	Adults
		2. Tattoo-based monitoring system.	[24]	9	34.78 ± 7.49	Adults
		3. Soft electrode array-based REM sleep stage monitoring system.	[43]	50	56.6 ± 8.4 (healthy) + (65.4 ± 7.6) Parkinson’s disease	Elderly
		4. At-home wireless sleep-monitoring patches.	[44]	-	-	-
4	Ear-EEG sleep-monitoring plugs and patches	1. In-ear viscoelastic earpiece.	[45]	5	28–25	Adults
			[46]	4	25, 28, 32, and 36	Adults
			[47]	4	25–36	Adults
			[48]	36	28.5 ± 5.3	Adults
			[49]	23	28.5 ± 5.3	Adults
			[50]	21	23.8 ± 4.8 years	Adults
		2. Wet electrode-based in-ear hardshell earpiece.	[51]	13	23–43	Adults
			[52]	9	26–44	Adults
			[53]	1	30	Adult
		3. Dry electrode-based in-ear hardshell earpiece.	[54]	20	22–36	Adults
		4. cEEGrid: flex-printed ear-EEG.	[55]	12	23–47	Adults
			[56]	20	34.9 ± 13.8	Adults
			[57]	10	28.4 ± 4.3	Adults
		5. Generic ear-EEG.	[58]	10	22–35	Adults
